# The use of systematic reviews in the planning, design and conduct of randomised trials: a retrospective cohort of NIHR HTA funded trials

**DOI:** 10.1186/1471-2288-13-50

**Published:** 2013-03-25

**Authors:** Ashley P Jones, Elizabeth Conroy, Paula R Williamson, Mike Clarke, Carrol Gamble

**Affiliations:** 1 Department of Biostatistics, Faculty of Health & Life Sciences University of Liverpool, Brownlow Street, Liverpool, L69 3GS, UK; 2 All Ireland Hub for Trials Methodology Research, Centre for Public Health, School of Medicine, Dentistry and Biomedical Sciences, Queens University Belfast, Royal Hospitals, Grosvenor Road, Belfast, BT12 6BJ, UK

**Keywords:** Systematic review, Meta-analysis, Randomised controlled trial, Planning, Design

## Abstract

**Background:**

A systematic review, with or without a meta-analysis, should be undertaken to determine if the research question of interest has already been answered before a new trial begins. There has been limited research on how systematic reviews are used within the design of new trials, the aims of this study were to investigate how systematic reviews of earlier trials are used in the planning and design of new randomised trials.

**Methods:**

Documentation from the application process for all randomised trials funded by the National Institute for Health Research Health Technology Assessment (NIHR HTA) between 2006 and 2008 were obtained. This included the: commissioning brief (if appropriate), outline application, minutes of the Board meeting in which the outline application was discussed, full application, detailed project description, referee comments, investigator response to referee comments, Board minutes on the full application and the trial protocol. Data were extracted on references to systematic reviews and how any such reviews had been used in the planning and design of the trial.

**Results:**

50 randomised trials were funded by NIHR HTA during this period and documentation was available for 48 of these. The cohort was predominately individually randomised parallel trials aiming to detect superiority between two treatments for a single primary outcome. 37 trials (77.1%) referenced a systematic review within the application and 20 of these (i.e. 41.7% of the total) used information contained in the systematic review in the design or planning of the new trial. The main areas in which systematic reviews were used were in the selection or definition of an outcome to be measured in the trial (7 of 37, 18.9%), the sample size calculation (7, 18.9%), the duration of follow up (8, 21.6%) and the approach to describing adverse events (9, 24.3%). Boards did not comment on the presence/absence or use of systematic reviews in any application.

**Conclusions:**

Systematic reviews were referenced in most funded applications but just over half of these used the review to inform the design. There is an expectation from funders that applicants will use a systematic review to justify the need for a new trial but no expectation regarding further use of a systematic review to aid planning and design of the trial. Guidelines for applicants and funders should be developed to promote the use of systematic reviews in the design and planning of randomised trials, to optimise delivery of new studies informed by the most up-to-date evidence base and to minimise waste in research.

## Background

The value of systematic reviews to identify areas requiring further health care research is well known [1], and a systematic review, with or without a meta-analysis, should be undertaken to determine if the research question of interest has already been answered before a new trial begins [2].

To help researchers use findings from systematic reviews in the design of new trials, a four stage framework has been suggested [3]: step one is to outline the populations, interventions, comparators, outcomes, timing and setting of the new trial; step two is to identify a relevant, valid and current systematic review; step three is to use the systematic review to inform the proposed trial; and step four is to summarise the implications for the new trial. However, there has been limited research on how systematic reviews are used within the design of new trials, with research to date focused on what is reported when the findings of a trial are published [4-6]. Given the potential for poorly planned trials to waste limited resources and introduce unnecessary delays in resolving the uncertainty that prompted the trial then every effort should be made to learn from the experience of relevant trials.

When publishing the results of a new trial the emphasis is on the ethical, scientific and environmental justification for conducting the trial and on setting its results in the context of the existing evidence base. It is therefore likely that trial publications may under report the ways in which systematic reviews were used to inform design. To overcome this limitation, we investigated a cohort of funding applications for randomised trials to identify where existing research is used not only to justify the need for conducting a trial but also to inform the design of the trial.

## Methods

We examined a cohort of randomised trials that had received funding from the National Institute for Health Research (NIHR) Health Technology Assessment (HTA) programme in the United Kingdom, in 2006, 2007 and 2008 due to the availability of the information that was required for the project. The NIHR HTA funding process (http://www.hta.ac.uk [accessed 30 April 2012]) involves several stages and all relevant documentation was provided: the commissioning brief (if appropriate), outline application, minutes of the Board meeting in which the outline application was discussed, full application, detailed project description, referee comments, investigator response to referee comments, Board minutes on the full application and the trial protocol.

A data extraction form was designed and piloted. Data were extracted to characterise the cohort and to categorise ways in which systematic reviews were used to inform the design of the proposed trial including the justification of treatment comparison, choice of frequency or dose, selection of (or definition of) an outcome, recruitment and consent rates, sample size (margin of equivalence or non-inferiority, size of difference, control group event rate, measure of variability and loss to follow up adjustment), length of follow-up, withdrawals, missing data or adverse events. Data on each application were extracted independently by two reviewers (APJ and EC). Disagreements were resolved by discussion or consultation with a third person (CG).

All results are presented in tables with number of occurrences and percentages; no formal statistical testing was undertaken. A confidentiality agreement with the NIHR HTA programme was signed prior to receiving the documentation for the cohort therefore the raw data cannot be made publicly available. Text extracts from the applications have been anonymised by removal of treatment or condition identifiers. Deleted text is denoted by *[…]* and *[words]* denotes addition of words or replaced words to aid understanding while maintaining anonymity.

## Results

We received documentation for the applications of 50 subsequently funded randomised trials, but there was insufficient documentation to consider two of these for inclusion in this study.

The characteristics of the 48 applications in the cohort are described in Table 1. The cohort was predominately individually randomised parallel trials aiming to detect superiority between two treatments for a single primary outcome with a median total sample size of 583. Table 2 describes the clinical area that the application was based in, with mental health and cancer having the most applications.

**Table 1:** Application characteristics

		**Number of trials**
		**(n=48), n (%)**
Type of trial	Parallel	42 (87.5)
	Cluster	5 (10.4)
	Factorial	1 (2.1)
Nature	Superiority	37 (77.1)
	Non-inferiority	7 (14.6)
	Equivalence	4 (8.3)
Number of Primary Outcomes	1	35 (72.9)
	2	9 (18.8)
	3	3 (6.3)
	4	1 (2.1)
Number of trial arms	2	37 (77.1)
	3	9 (18.8)
	4	2 (4.2)
Median Sample size (range)	583 (172 to 20,000)	

**Table 2:** Clinical application of applications

**Clinical area**	**Number of applications**
	**N=48**
Alcohol consumption	1
Antibiotic associated diarrhoea	1
Bowel problems	1
Cancer	6
Diabetes/Hyperglycaemia	2
Falls in the elderly	1
Haemorrhage/blood and vessel/vein disorders	5
Heart/respiratory	5
Infections	3
Mental health	10
Musculoskeletal	4
Pregnancy/Labour	1
Skin conditions	2
Sleep disorders/sedation/anaesthesia	2
Stroke	2
Urinary problems/kidneys	2

Eleven (21%) applications made no reference to a review and 37 applications (79%) referenced a systematic review within the application for funding. Twenty of these 37 applications (54.1%) reported the use of the systematic review in the design of the RCT, the other 17 did not.

### Applications that did not reference a systematic review

Seven of the eleven applications that did not reference a systematic review stated that there had been no previous trials either of the interventions to be compared or within the relevant population, with one of these explicitly stating an absence of a systematic review. However, it was not clear within these seven cases that the absence of relevant studies was based on a robust search strategy. One of the other applications was for a pilot study implying the absence of previous relevant trials, one discussed an on-going study, and two discussed relevant randomised trials but with no clear indication of how these had been identified. For all eleven of these applications, the absence of a systematic review was not referred to in the Board minutes or the referees’ comments.

### Applications that did reference a systematic review

Of the 37 applications that did reference a systematic review, 15 referenced more than one systematic review, with 65 systematic reviews referenced in total across the applications. Twenty three (35%) of these 65 systematic reviews had been published in the Cochrane Database of Systematic Reviews and 42 (65%) had been published in another journal. Twenty (54.1% of the 37) of the applications used the systematic review in some way in the design or planning of the new trial (Table 3) with the median (range) number of ways a review was used in each of these 20 applications being 2 (1 to 5).

**Table 3:** The use of systematic reviews in trial design

**Area of use**	**Number of applications (%)**
	**(n=37)**
Justification of treatment comparisons	6 (16.2)
Choice of frequency/dose	2 (5.4)
Selection or definition of outcome	7 (18.9)
Recruitment and consent	2 (5.4)
Estimating the difference to detect or margin of equivalence	6 (16.2)
Estimating the control group event rate	3 (8.1)
Inform standard deviation	1 (2.7)
Duration of follow up	8 (21.6)
Withdrawal rate	1 (2.7)
Adverse events	9 (24.3)

### Justification of treatment comparisons

Six applications used systematic reviews in the justification of the treatment group (see Additional file 1).

Five of these six applications justified the proposed treatments based on effectiveness shown in a systematic review.

In response to a referee’s comment to ‘make a stronger case for this RCT’ and justify the chosen treatment regimes, applicants made greater use of evidence from a Cochrane Review initially referenced in their application (application 5). In another application (application 6), a referee requested justification for participant inclusion criteria and the applicants responded highlighting data from a trial which was included within the systematic review.

### Choice of frequency/dose

Two applications used a systematic review in the choice of dose of the intervention (see Additional file 2). One application used essentially the same dose as one of the trials in the systematic review but stated the frequency of administration had not been previously tested in a trial (application 7). The second application stated that the systematic review showed that dosing regimens varied widely and the dose range that was chosen for the new trial was based on one within that range that had shown clinical benefit (application 8).

### Selection or definition of an outcome

Seven applications used evidence from systematic reviews to justify the outcome in the proposed trial (see Additional file 3).

In five of the seven applications the systematic review had specifically stated the outcome or outcomes that should be measured in future research and this was used as justification for choosing a specific outcome in the applications.

In one application a systematic review was used to identify previous commonly used measures of health-related quality of life, but the application did not intend to use those identified within the systematic review and provided justification for this (application 10).

One application used the systematic review to inform the definition of a secondary outcome of the study and the cut off for the outcome in the application was based on evidence from the systematic review (application 13).

### Recruitment and consent

Two applications used information presented in systematic reviews to aid planning of patient recruitment (see Additional file 4).

Only one application explicitly discussed consent rates as observed within trials included within the systematic review. This application justified the consent rate assumed in the application based on relevant previous trials in the systematic review (application 2).

The second application used poor recruitment rates reported in previous relevant trials included in a systematic review to determine site selection criteria (application 10).

### Sample size calculation

#### Estimating the difference to detect or the margin of equivalence

Five of the 37 (13.5%) applications that aimed to detect a difference referenced a systematic review when defining the size of the difference that they wished to detect in their trial. The applications used the actual difference from the meta-analysis or the applicants used a figure from one of the trials in the systematic review (see Additional file 5).

In one (9.1%) of the eleven equivalence/non-inferiority trials, a systematic review was referenced when defining the margin of equivalence. The applicants stated that they used the effect estimate from the systematic review as the margin of equivalence. However, we were unable to determine which review this data came from when we checked the four reviews referenced in the application (application 4).

#### Estimating the control group event rate

Three applications used a systematic review to estimate the control group event rate (see Additional file 6). The figures that were used in the sample size calculations were taken directly from the systematic review in two examples (application 7 and 9) and, in the third example, the figures were very similar to those in the review (application 14).

#### Inform standard deviation

One application referenced a systematic review to justify the standard deviation that they had used in the sample size calculation; the standard deviation that was used was the mid-point of the standard deviations of the two trials included in the review (see Additional file 7).

### Duration of follow up

Eight applications referred to a systematic review in order to justify the duration of follow up during a trial (see Additional file 8).

Based on the conclusions within systematic reviews, six applications called for long term follow up, rather than short term follow up and two applications stated that the systematic review supported establishing evidence for both short term and long term effects (applications 16 and 20). We found no definitions of ‘long term’ or ‘short term’ within the recommendations of any of these systematic reviews.

### Withdrawal rate

One application increased their sample size by a fixed amount based on estimates of the withdrawal rate from trials in the systematic review (see Additional file 9).

### Adverse events

Nine applications provided information on adverse events that had been reported from a systematic review (see Additional file 10). In two applications, the number of events and rates were described in detail (applications 8 and 19). In six applications it was reported on whether the treatment(s) had shown any increase or decrease in adverse events and in one application (application 20) it was reported that the systematic review had recommended investigation possible adverse effects of the drug.

## Discussion

This study has investigated how systematic reviews were used in the planning and design of randomised trials that have been funded by the NIHR HTA programme during the period of 2006 to 2008. The results show that systematic reviews are referenced in the majority (77.1%) of successful applications for funding of randomised trials by the HTA, but 17 of these 37 applications (45.9%) did not report the use of the systematic review in the design of the trial. The main areas in which systematic reviews were used in the planning of the new trial were in the selection or definition of the outcome, the sample size calculation and the duration of follow up. Descriptions of adverse events in previous studies were referenced, but it was unclear whether they were then used to inform the study adverse event planning.

There is limited evidence on how systematic reviews are used in the planning and design of randomised trials. This study is unique both in terms of the number of trials included and the information that was reviewed, because all documentation from the application process was made available to us. One potential weakness of this study was that we were not permitted to have access to unsuccessful applications, meaning that we are not able to assess if there is a difference in the use of systematic reviews between those applications that were successful in gaining funding and those that were not. This work has focused on a single UK funding body which funds trials across a range of health care settings and conditions. We are not aware of any reasons why the results from the study will not be generalisable to other funding agencies and to other countries.

Previous research has shown that authors of new randomised trials were often unaware of relevant reviews when designing trials. For example, Cooper et al. [6] found that 13 (55%) of 24 authors of new published trials included in an updated Cochrane Review were unaware of the review version available at the time of study design and eight (33%) used the systematic review in study design. Goudie et al. [4] looked at how trials were designed and reported in the context of previous evidence in two journals (JAMA and the Archives of Internal Medicine) and reported that previous trial results were consulted during the design of 10 trials (37%). This phenomenon of researchers not consulting previous evidence was highlighted by Clarke et al. who stated that most researchers ‘do not seem to have considered systematic reviews when designing their trial’ [5]. Limitations on the available resources to conduct clinical trials, both in patient populations and costs, would suggest that every effort should be made to learn from the experience of relevant trials.

There are over 2500 systematic reviews published every year (a quarter of which are on the Cochrane Database of Systematic Reviews [7]). It is essential that researchers applying for funding for new trials search the literature to identify relevant systematic reviews and use that information not only to justify the need for the new trial but also to design and plan that trial [8]. There are also calls for authors of reviews to be more specific about what further research is needed [1,9].

It is important that funders of trials ensure that applicants have used the most up-to-date and relevant evidence in designing a trial and that information on how the evidence has been used is described in the application. We recommend that applicants stating absence of previously conducted relevant trials support this statement by providing a systematic search strategy, and that funding bodies ask applicants to justify the trial design in each of the areas listed within Table 2. These are details that could be requested by the CONSORT statement in the reporting of a trial [10]. The potential for systematic reviews to impact on trial design could be maximised if authors of systematic reviews are more specific about what further research is needed.

The PRISMA (Preferred Reporting Items for Systematic reviews and Meta-Analyses) checklist can be included when reporting a systematic review and item #26 does state that authors should ‘Provide a general interpretation of the results in the context of other evidence, and implications for future research’ [11], however we believe there should be a minimum set of recommendations within the implications for future research and that these should include recommendations for the types of intervention, participants and outcomes to be studied.

### Future research

Our study has highlighted that the majority of new NIHR HTA-funded trials referenced systematic reviews during the application process, to justify the new trial but that even among those that did reference a systematic review, almost half did not use the review to inform the trial design. We plan to investigate guidelines from organisations that fund randomised trials to gather details of the information that is requested when funding applications are made. The use of systematic reviews within Value of Information (VOI) analyses, which aim to provide an objective method for informing the decision of which trials to fund and how best to design them, should also be explored.

## Conclusions

Systematic reviews are frequently referenced in the majority of successful applications for funding but underutilised in the planning of that research. There is an expectation from funders that applicants will use a systematic review to justify the need for a new trial but no clear expectations regarding further use of a systematic review to aid planning and design of the trial. Guidelines for applicants and funders should be developed to promote the use of systematic reviews in the design and planning of randomised trials, to optimise delivery of new studies informed by the most up-to-date evidence base and to minimise waste in research. Systematic review authors could maximise the impact on trial design by reporting the factors that are important in planning a future trial.

## Competing interests

The authors declare that they have no competing interests.

## Authors’ contributions

PRW and MC were involved in the design of the study and commented on the drafts of the paper. CG was involved in the design of the study, analysed the results and co-wrote the paper. AJ was involved in the design of the study, extracted the data, analysed the results and co-wrote the paper. EC extracted the data and commented on drafts of the paper. All authors read and approved the final manuscript.

## Pre-publication history

The pre-publication history for this paper can be accessed here:

http://www.biomedcentral.com/1471-2288/13/50/prepub

## Supplementary Material

Additional file 1 How an application used a systematic review for the justification of treatment comparisons. Click here for file

Additional file 2 How an application used a systematic review for the choice of the frequency/dose. Click here for file

Additional file 3 How an application used a systematic review for the selection or definition of an outcome. Click here for file

Additional file 4 How an application used a systematic review for recruitment and consent. Click here for file

Additional file 5 How an application used a systematic review in estimating the difference to detect or margin of equivalence. Click here for file

Additional file 6 How an application used a systematic review to estimate the control group event rate. Click here for file

Additional file 7 How an application used a systematic review to inform the standard deviation. Click here for file

Additional file 8 How an application used a systematic review to justify the duration of follow up. Click here for file

Additional file 9 How an application used a systematic review to estimate withdrawal rate. Click here for file

Additional file 10 How an application used a systematic review to describe adverse events. Click here for file
