# Supplementary material for: The use of systematic reviews in the planning, design and conduct of randomised trials: a retrospective cohort of NIHR HTA funded trials
Source: BMC Med Res Methodol. 2013 Mar 25;13:50. doi: 10.1186/1471-2288-13-50 (PMC3621166; doi:10.1186/1471-2288-13-50)
Supplement: Additional file 1 — How an application used a systematic review for the justification of treatment comparisons. [file 1471-2288-13-50-S1.docx]

Table 1: How applications used systematic review for justification of treatment comparisons

| Application | Statement |
| --- | --- |
| 1 | We conclude that *[based on a meta-analysis]* there is currently inadequate evidence to support *[this treatment]* as standard treatment. A large trial would allow head-to-head comparison *[...]* as well as comparing each treatment to standard therapy. |
| 2 | A number of systematic and meta-reviews have highlighted the potential efficacy of *[treatment 1]* in the prevention and treatment of *[condition 1].* Although these reviews have been positive a number of issues have been identified including the absence of comparisons with appropriate placebo groups, the need for longer term follow-up and the absence of randomised controlled trials within a UK context. |
| 3 | A ‘no treatment’ arm will not be included in this study. There are a number of reasons for this. Firstly, the systematic review showed that *[treatment 1]* is more effective than ‘no treatment’ whilst failing to find any evidence for the effectiveness of *[treatment 2]*. Therefore, the important clinical question is whether the use of *[treatment 2]* is superior to that of the standard effective treatment. |
| 4 | We propose, based on our systematic review that *[treatment 1]* is given in *[this condition]* only if *[treatment 2]* has failed. |
| 5 | Referee’s comment:  ‘I feel that the investigators could make a stronger case for this RCT. I would have liked an explicit explanation of why the investigators expect that *[treatment 1]* outcomes are different given that the majority of patients are currently treated *[using treatment 2]*. They only report that there is insufficient evidence to support *[treatment 1]* according to a Cochrane review’. The investigators state that they are going to produce patient information material that will provide a balanced account of the possible benefits and risk of the interventions under test. They might want to include this account in their current application. This would help referees who are no experts in this field to evaluate the need for a trial and the existence of equipoise.  *Applicant’s response:*  ‘To illustrate this, we have used data from randomised trials presented in our Cochrane review. While we are considering *[condition 1]*, *[trial 1]* showed *[outcome 1]* at 1 and 2 years after *[condition 1]*. For instance, at 2 years: *[a]* % had *[outcome 1]*; *[b]* % had *[outcome 2]*; *[c]* % had *[outcome 3]*; and *[d]* % *[had outcome 4]*. *[Trial 2]* included participants with *[condition 2]* and found that, for instance, at 1 year: *[e]* % *[had outcome 5]*; and *[f]* % *[had outcome 6]*.’ |
| 6 | Referee Comment:  ‘Previous studies referred to in the application (*[study1]* and *[study 2]*) found no effect *[...]* in *[this age group with this condition 1].* Can the applicants justify the inclusion of *[this age subgroup]* who have *[condition 2 and condition 1]?’*  Applicant’s Response:  ‘We have excluded severe *[condition 1]* as specified in the original HTA call. We have not excluded all *[levels of condition 1]* in order to keep inclusion criteria as broad as possible to enhance the `generalisability’ of any findings. The *[study 1]* referred to offered only *[...]* brief intervention which the authors indicate may not be enough for *[this subgroup with condition 1]*. Our study will offer more intensive treatment. The authors also note that statistical power of some analyses relating to the *[age group with and without condition 1]* was limited by sample size. Other studies, powered to detect changes in condition 1, rather than condition 2 have shown a positive impact on condition 1. On the basis of these mixed findings there is no strong case to exclude *[this age group with condition 1]* from the study.’ |
