# Supplementary material for: The use of systematic reviews in the planning, design and conduct of randomised trials: a retrospective cohort of NIHR HTA funded trials
Source: BMC Med Res Methodol. 2013 Mar 25;13:50. doi: 10.1186/1471-2288-13-50 (PMC3621166; doi:10.1186/1471-2288-13-50)
Supplement: Additional file 2 — How an application used a systematic review for the choice of the frequency/dose. [file 1471-2288-13-50-S2.docx]

Table 1: How applications used systematic review for choice of the frequency/dose

| Application | Statement |
| --- | --- |
| 7 | We have used essentially the same dose as *[a previous study] [...]*. However, we will be using three doses in an hour, which is different from previous paediatric studies. |
| 8 | The systematic review of randomised controlled trials of *[treatment1] [...]* showed that dose regimens *[...]* vary widely. Therefore a fixed dose within the dose range which has been shown to *[…]* provide clinical benefit has been selected. |
