# Supplementary material for: The use of systematic reviews in the planning, design and conduct of randomised trials: a retrospective cohort of NIHR HTA funded trials
Source: BMC Med Res Methodol. 2013 Mar 25;13:50. doi: 10.1186/1471-2288-13-50 (PMC3621166; doi:10.1186/1471-2288-13-50)
Supplement: Additional file 3 — How an application used a systematic review for the selection or definition of an outcome. [file 1471-2288-13-50-S3.docx]

Table 1: How applications used systematic review for selection or definition of an outcome

| Application | Statement |
| --- | --- |
| 1 | Previous studies (outlined in the meta-analysis) have used measures of *[this endpoint*], such as *[outcome 1],* as their primary outcome. In some studies these have shown that treatment with *[treatment 1 or treatment 2]* may be associated with significant changes in *[outcome 1]*. However, it is not clear whether these changes lead to important changes in patient management or a clinically meaningful improvement in symptoms.  We have selected two primary outcomes to identify important changes in patient management and symptoms *[of this condition].* These outcomes have been chosen after literature review and consultation with our consumer representatives, and reflect health service and patient perspectives respectively. |
| 5 | The methodology underlying our proposal has been particularly informed by issues raised from the critical appraisal of trials in this and related areas. These include the need for good quality methodology, such as independent randomisation and active and systematic methods of follow-up that by minimising bias will generate reliable evidence. More subject specific issues include an appreciation of problems surrounding fracture classification *[Trial A; Trial B],* the need for validated and, moreover, patient-centred functional outcome assessment, and for sufficiently long-term follow up. |
| 9 | In summary, the majority of clinical trials conducted thus far have been small and of poor to moderate quality in terms of trial methodology and design, outcome measures (such as reporting of *[outcome 1]* rather than *[outcome 2]*) and the lack of a comprehensive evaluation. |
| 10 | The HTA commissioned a systematic review into outcome measures for *[condition 1]*. At that time the three measures of health-related quality of life that had been most commonly used in follow-up studies of *[condition 1]* were the SIP/FLP (Sickness Impact Profile/Functional Limitations Profile), the PQOL (Perceived Quality of Life) and the NHP (Nottingham Health Profile). In addition, the SF-36 (Short Form 36 Health Survey Questionnaire) was increasingly being used.  The decision to use EQ-5D in *[this]* study has been made on a number of grounds. The EQ-5D serves both as a measure of health-related quality of life and as a utility measure for calculating quality-adjusted life years. There is a large (3,400) reference population database available, and [study A] will generate data on a population of mixed ICU survivors at the same time as [this study] is running, so we will have two appropriate reference populations. There is a large 11 centre study of *[patients]* planned in *[the]* USA which will use EQ-5D as an outcome measure, allowing trans-Atlantic comparisons. |
| 11 | Importantly, *[the systematic review]* also advocated the need for research on the long term impact on QoL and cost effectiveness of *[the treatment]*. |
| 12 | In addition *[the systematic review]* identified the need for trials to use instruments to measure outcome which have been validated for use in *[this condition].* |
| 13 | This cut-off was frequently used amongst trials included in the systematic review of the effectiveness and is also being used in other trials currently underway *[...]*. |
