# Supplementary material for: The use of systematic reviews in the planning, design and conduct of randomised trials: a retrospective cohort of NIHR HTA funded trials
Source: BMC Med Res Methodol. 2013 Mar 25;13:50. doi: 10.1186/1471-2288-13-50 (PMC3621166; doi:10.1186/1471-2288-13-50)
Supplement: Additional file 4 — How an application used a systematic review for recruitment and consent. [file 1471-2288-13-50-S4.docx]

Table 1: How an application used a systematic review for recruitment and consent

| Application | Statement |
| --- | --- |
| 2 | *[...]* programmes tend to achieve recruitment rates of 67-88%. *[…]*our predictions for this study are more conservative and we predict a recruitment rate of 70%. |
| 10 | To run a randomised controlled trial of *[device 1]* in *[sites]* that already own *[device 1]* would require all clinicians caring for the patients to be substantially uncertain about which *[device]* was best for their patients. We tried to find evidence in the literature to determine whether these problems (case selection and cross over from control to treatment group) had occurred in previous trials *[…]*. These data suggest marked case selection was taking place *[…]*. Based on the data and arguments above, we believe there are considerable risks to running the trial in centres which already have *[device 1]*. |
