# Supplementary material for: The use of systematic reviews in the planning, design and conduct of randomised trials: a retrospective cohort of NIHR HTA funded trials
Source: BMC Med Res Methodol. 2013 Mar 25;13:50. doi: 10.1186/1471-2288-13-50 (PMC3621166; doi:10.1186/1471-2288-13-50)
Supplement: Additional file 5 — How an application used a systematic review in estimating the difference to detect or margin of equivalence. [file 1471-2288-13-50-S5.docx]

Table 1: How an application used a systematic review in estimating the difference to detect or margin of equivalence.

| Application | Statement |
| --- | --- |
| 3 | The *[...]* systematic review found only one small trial directly comparing the effectiveness of *[the interventions]*. This poor quality study found a 58% cure rate among the patients allocated to *[treatment 1]* compared with 41% among those treated with *[treatment 2]*. This difference of 17% was not statistically significant. In this study *[of treatment 1 vs treatment 2]* we have decided to power the trial to show a 15% difference in effectiveness. |
| 4 | Change in *[outcome 1]* was reported in 8 RCTs in the established *[…]* systematic review. The minimal clinically important *[change in outcome 1]* in RCTs is 0.22 *[...]*.  The minimally clinically important change in *[outcome 1]* is considered to be 0.22 *[...]* The trial will therefore be designed under the assumption that *[treatment 1 and treatment 2]* produce equivalent reductions in *[outcome 1]* and that a difference of less than 0.22 will be regarded as equivalence. |
| 10 | To detect a minimal, clinically important, absolute risk reduction of 8% (half that observed in the largest previous RCT). |
| 13 | Therefore, we have based our expected treatment effect upon systematic review evidence for the effectiveness of *[treatment 1]*. This review concluded that there was a three-fold increased odds of *[outcome 1]* for *[treatment 1]* compared with *[treatment 2]* .However, as this review did not focus specifically on patients with *[condition 1]*, we expect a reduced effect and have therefore based our sample size calculation on detecting an odds ratio of 2. |
| 14 | In keeping with the meta-analyses quoted above, we predict that the *[intervention]* will reduce the frequency of *[the event]* by 50% (i.e. to 10%). |
| 15 | ...... we note that the mean effect size in RCTs identified by the *[...]* systematic review in the area was 0.5% (0.33SD). We propose to recruit sufficient *[...]* to allow the trial to detect a difference between groups of 0.5 standard deviations.  Referee’s comment:  ‘The justification for the chosen effect size seems somewhat unsure when it is compared to the effect size of *[trial 1]*, which showed an effect that was twice as large. However, some information is provided to suggest that smaller effect sizes may also be relevant.’  Applicant’s response:  ‘There is evidence (presented) that smaller effect sizes than that found in the *[trial 1]* are clinically very relevant. Powering the trial to be able to detect an effect size similar to that found in *[trial 1]* would lead to the possibility of having inadequate power to detect an effect that could well be clinically highly significant.’ |
