# Supplementary material for: The use of systematic reviews in the planning, design and conduct of randomised trials: a retrospective cohort of NIHR HTA funded trials
Source: BMC Med Res Methodol. 2013 Mar 25;13:50. doi: 10.1186/1471-2288-13-50 (PMC3621166; doi:10.1186/1471-2288-13-50)
Supplement: Additional file 6 — How an application used a systematic review to estimate the control group event rate. [file 1471-2288-13-50-S6.docx]

Table 1: How an application used a systematic review to estimate the control group event rate.

| Application | Statement |
| --- | --- |
| 3 | The overall cure rates from this study are smaller than those observed in two placebo controlled trials of *[treatment 1]*, both of which reported cure rates of 85% for active treatment, possibly because more resistant *[conditions]* were included in the study comparing *[treatment 2]* with *[treatment 1]*. In this study we have decided to power the trial to show a 15% difference in effectiveness. We therefore, will recruit sufficient patients to give us 80% power (5% two sided significance) to show a difference in cure rates of 70% versus 85%. |
| 9 | Based on the Cochrane review and other data, the anticipated incidence of *[outcome 1]* in the standard control group is 7% and it is reasonable to hypothesise that [*treatment 1*] will reduce this to about 4%. |
| 14 | A combined event rate *[outcome 1]* (21.5%) was used to estimate the occurrence *[of outcome 2]* as 20% for the sample size calculation. |
