# Supplementary material for: The use of systematic reviews in the planning, design and conduct of randomised trials: a retrospective cohort of NIHR HTA funded trials
Source: BMC Med Res Methodol. 2013 Mar 25;13:50. doi: 10.1186/1471-2288-13-50 (PMC3621166; doi:10.1186/1471-2288-13-50)
Supplement: Additional file 7 — How an application used a systematic review to inform the standard deviation. [file 1471-2288-13-50-S7.docx]

Table 1: How an application used a systematic review to inform the standard deviation

| Application | Statement |
| --- | --- |
| 4 | We have taken the average SD for changes in *[outcome 1]* in these two trials. |
