# Supplementary material for: The use of systematic reviews in the planning, design and conduct of randomised trials: a retrospective cohort of NIHR HTA funded trials
Source: BMC Med Res Methodol. 2013 Mar 25;13:50. doi: 10.1186/1471-2288-13-50 (PMC3621166; doi:10.1186/1471-2288-13-50)
Supplement: Additional file 8 — How an application used a systematic review to justify the duration of follow up. [file 1471-2288-13-50-S8.docx]

Table 1: How an application used a systematic review to justify the duration of follow up.

| Application | Statement |
| --- | --- |
| 2 | The absence of long term follow-up and the tendency for post-intervention effects *[...]* to diminish after 6 months have been noted. |
| 5 | Based on existing evidence there is a need for sufficiently long term follow up**.** |
| 11 | A recent Cochrane review concluded that there was limited evidence that *[treatment 1]* was more effective than standard treatment *[...]* and that long term benefits were unclear. |
| 12 | *[the authors of a trial included in the systematic review]* acknowledged the need for research into *[...]* longer-term treatment. |
| 16 | Only one of the trials studied whether any benefits of *[the intervention]* outlasted the duration of the intervention. In the meta-regression the length of follow-up was an important source of heterogeneity, with those of shortest duration reporting the largest effects suggesting that effects may be weak or non-existent over the long-term. In the context of a chronic relapsing and remitting disease it is important to estimate the long-term as well as the short-term effects, though even a short term benefit may still be cost-effective.’ |
| 17 | Unfortunately, the long term effectiveness *[treatment 1]* has not been rigorously established as these studies *[in the systematic review]* were small and only had short follow-up limited to a few months. |
| 18 | Although the literature supports the short-term efficacy of *[treatment 1]* as an effective alternative to conventional *[treatment],* long-term data are lacking. |
| 20 | Study should evaluate short term and long term effects of drug and possible AE on *[condition A].* |
