# Supplementary material for: The use of systematic reviews in the planning, design and conduct of randomised trials: a retrospective cohort of NIHR HTA funded trials
Source: BMC Med Res Methodol. 2013 Mar 25;13:50. doi: 10.1186/1471-2288-13-50 (PMC3621166; doi:10.1186/1471-2288-13-50)
Supplement: Additional file 9 — How an application used a systematic review to estimate withdrawal rate. [file 1471-2288-13-50-S9.docx]

Table 1: How an application used a systematic review to estimate withdrawal rate.

| Application | Statement |
| --- | --- |
| 3 | There have been very few good quality trials on which to base the likely dropout rates. The overall reported dropout rates in the two trials *[directly comparing the two treatments]* were 6.5% and 35% whilst several trials *[which used the control]* reported dropout rates of around 10%. We have therefore decided to allow a 10% loss of follow up-up in this study. |
