# Supplementary material for: The use of systematic reviews in the planning, design and conduct of randomised trials: a retrospective cohort of NIHR HTA funded trials
Source: BMC Med Res Methodol. 2013 Mar 25;13:50. doi: 10.1186/1471-2288-13-50 (PMC3621166; doi:10.1186/1471-2288-13-50)
Supplement: Additional file 10 — How an application used a systematic review to describe adverse events. [file 1471-2288-13-50-S10.docx]

Table 1: How an application used a systematic review to describe adverse events.

| Application | Statement |
| --- | --- |
| 3 | Many patients experience unpleasant side effects such as pain and blistering during *[treatment1].* |
| 4 | Systematic reviews of *[treatment 1]* show some increase in toxicity over *[treatment 2].* The early review *[...]* concluded risks exceeded benefits but recent reviews suggest the balance favours *[treatment 1]*. |
| 7 | Most importantly there were no adverse events reported and so the other important conclusion was that *[treatment 1]* was safe. |
| 8 | The numbers of adverse events in individual studies were small but there was no evidence of an increased risk of *[adverse event 1]* (4 events treatment versus 6 events control), *[adverse event 2]* (8 events treatment versus 2 events control), *[adverse event 3]* (6 events treatment versus 8 events control) or *[adverse event 4]* (0 events treatment versus 4 events control). The included trials were not large enough to exclude rare adverse reactions but *[treatment 1]* has been used for many years and serious toxicity seems unlikely. |
| 12 | One study of *[treatment 1]* indicated that active treatment increased *[outcome 1 and outcome 2]*, whilst several studies of *[outcome 3]* indicate that most *[treatments]* increase *[the risk of outcome 3]*. |
| 17 | Each of these studies demonstrated small improvements in *[outcome 1]* with no increase in *[three other outcomes].* |
| 18 | *[The systematic review]* concluded that *[treatment 1]* appeared to be safe. |
| 19 | *[Treatment 1]* had more severe adverse effects (including a death *[...]*) and disease recurrence. Most of the adverse effects in *[treatment 2]* were mild. |
| 20 | Study should evaluate short term and long term effects of drug and possible AE on [condition A]. |
